# Diversity Considerations for Promoting Early Childhood Oral Health: A Pilot Study

**DOI:** 10.1155/2014/175084

**Published:** 2014-01-30

**Authors:** Sarah Prowse, Robert J. Schroth, Alexandria Wilson, Jeanette M. Edwards, Janet Sarson, Jeremy A. Levi, Michael E. Moffatt

**Affiliations:** ^1^Department of Preventive Dental Science, University of Manitoba, 507–715 McDermot Avenue, Winnipeg, MB, Canada R3E 3P4; ^2^Winnipeg Regional Health Authority, 490 Hargrave Street, Winnipeg, MB, Canada R3A 0X7; ^3^The Manitoba Institute of Child Health, 715 McDermot Avenue, Winnipeg, MB, Canada R3E 3P4; ^4^College of Education, University of Saskatchewan, 28 Campus Drive, Saskatoon, SK, Canada S7N 0X1; ^5^Manitoba Health, 300 Carlton Street, Winnipeg, MB, Canada R3B 2K6; ^6^Department of Community Health Sciences, University of Manitoba, S113–750 Bannatyne Avenue, Winnipeg, MB, Canada R3E 0W3; ^7^Research & Applied Learning, Winnipeg Regional Health Authority, 650 Main Street, Winnipeg, MB, Canada R3B 1E2

## Abstract

*Objectives*. Several groups in Manitoba, Canada, experience early childhood caries (ECC), including Aboriginal, immigrant, and refugee children and those from select rural regions. The purpose of this pilot study was to explore the views of parents and caregivers from four cultural groups on early childhood oral health and ECC. *Methods*. A qualitative descriptive study design using focus groups recruited parents and caregivers from four cultural groups. Discussions were documented, audio-recorded, transcribed, and then analyzed for content based on themes. *Results*. Parents and caregivers identified several potential barriers to good oral health practice, including child's temperament, finances, and inability to control sugar intake. Both religion and genetics were found to influence perceptions of oral health. Misconceptions regarding breastfeeding and bottle use were present. One-on-one discussions, parental networks, and using laypeople from similar backgrounds were suggested methods to promote oral health. The immigrant and refugee participants placed emphasis on the use of visuals for those with language barriers while Hutterite participants suggested a health-education approach. *Conclusions*. These pilot study findings provide initial insight into the oral health-related knowledge and beliefs of these groups. This will help to inform planning of ECC prevention and research strategies, which can be tailored to specific populations.

## 1. Introduction

Oral health plays an important role in overall health. This is particularly true during early childhood as oral health can influence overall health and well-being [1]. Keeping primary teeth healthy is essential as those who suffer from caries in their preschool years are more likely to experience caries throughout childhood and adolescence [2, 3].

Early childhood caries (ECC) is decay affecting the primary dentition of children <72 months of age [4, 5]. Several groups have been found to be at a high risk for ECC including First Nations and Aboriginal children, refugees and newcomers, and those experiencing poverty [6–9]. Prevalence rates for ECC in several distinct Canadian pediatric populations have been reported with most groups exhibiting rates above 40%. For instance, urban and on-reserve First Nations and Aboriginal children are reported to have high rates, sometimes reaching 80–90% of the population with many meeting the definition of severe early childhood caries (S-ECC), a more rampant form of ECC [8–10]. Meanwhile nearly 40% of rural Hutterite children have been reported to have S-ECC [11]. Other groups in Canada such as Vietnamese children, immigrants from South Asia, and Portuguese-speaking immigrants have been reported to experience ECC [12–14].

However, still very little is known about the oral health of newcomers, although anecdotal conversations with practitioners would suggest a high level of dental needs. There is a growing realization that newcomers are at increased risk for caries as the American Academy of Pediatric Dentistry (AAPD) has included a question on “immigrant status” in their caries-risk assessment tool (CAT) [15].

There are many challenges involved in promoting oral health to high-risk groups. First and foremost is the difficulty associated with reaching these populations. Additionally, “one-size-fits-all” approaches and strategies that have worked with the general population often have little impact on reducing the incidence of ECC in high-risk populations and may not be effective with distinct cultural groups [16]. Even if it were possible to reach all high-risk children and provide them with tailored programming, the desired behavioural change may not take place (despite the increase in knowledge offered by traditional oral health approaches) [16].

Differing practices and views on oral health, which may be related to cultural diversity, may contribute to increased caries risk. Many aspects of cultural diversity can influence oral hygiene routines, diet, health beliefs, reaction to pain, and access to care, factors which may in turn affect oral health status [17, 18]. If a person belongs to a cultural group that does not define poor oral health as abnormal, they may lack both information about oral health and access to care and may not comply with professional recommendations for treatment [18, 19].

There is a growing realization that qualitative research methods are useful in identifying how knowledge and ideas “develop and operate within a given cultural context” [20]. Overall, there is limited qualitative research on the topic of ECC and the promotion of early childhood oral health (ECOH) among cultural minority groups in North America [21, 22]. There is a growing realization that qualitative research methods may be helpful to uncover family and cultural issues that influence infant and preschool oral health. Having an appreciation of different cultural views may allow for focused outreach and promotion activities [23, 24]. While known barriers to good oral health include a lack of funds to seek dental care (especially with newcomer populations [25]), the effect of knowledge and beliefs on child oral health is less well understood. Parental and caregiver lack of knowledge of and negative attitude towards preschool oral health have been found to be associated with increased caries experience in their young children [23].

The purpose of this pilot study was to examine the knowledge and beliefs of parents and caregivers from four different cultural groups with respect to ECOH and ECC. The ultimate goal was to use these findings to assist in tailoring ongoing promotional activities to improve ECOH and prevent ECC.

## 2. Methods

A qualitative study design using focus groups was chosen to explore parent and caregiver views on ECOH and ECC from four different cultural groups. This pilot study was undertaken by the Healthy Smile Happy Child (HSHC) partnership that has been promoting ECOH in Manitoba, Canada, since 2000. The partnership adopted and maintains a community engagement approach to address ECC and has been guided by three pillars: community development, health promotion and education and evaluation [26–28]. Focus groups were selected as health promotion programs can often be strengthened through participatory planning approaches that allow participants to voice their experiences and opinions [29]. The project team recognized the value of focus groups and the different findings that can be obtained using such an approach.

Four pilot focus groups involving parents and caregivers of children <6 years of age were held. Each focus group involved a different cultural group and was held in southern Manitoba, Canada. A nonprobabilistic approach to recruitment using a convenience sample of participants was selected. The four groups included parents and caregivers from an urban Aboriginal community, a rural Hutterite colony in southwest Manitoba, a refugee group in the city of Winnipeg, and an urban group of recent newcomer immigrants to the city of Winnipeg, Manitoba. These four distinct groups were selected as children from these communities often experience a higher burden of ECC than the mainstream population.

Aboriginal participants were recruited through an Aboriginal Head Start program and an organization providing culturally relevant preventive and supportive programming to families. All participants were self-identified as Aboriginal (First Nations or Métis). Hutterite participants were recruited with the help of a teacher and research assistant who was a member of a Hutterite colony and who had an existing working relationship with the Department of Pediatrics and Child Health at the University of Manitoba. The Hutterite live on colonies and are a communal branch of Anabaptists (like the Amish and Mennonites) [30]. Meanwhile, refugee participants were recruited through the Canadian Muslim Women's Institute. Participants had refugee status and had been in Canada for at least one year. Finally, the newcomer focus group participants were recruited from an English-as-an-additional-language (EAL) program in Winnipeg, Canada. Participants were landed immigrants, who had an English benchmark of at least four and who had been in Canada for at least one year.

The team facilitating the focus groups included a qualitative research consultant and a HSHC staff member. The study was approved by the University of Manitoba Health Research Ethics Board and followed established community research protocols. Participants provided written informed consent and permission for audiorecording of the discussions. The research team made notes on a flipchart during the discussions while the HSHC team member took additional notes. Participants were invited to review the notes and to correct, delete, or add to any inaccurate or inadequate representations of their comments. Participants in the urban focus groups were provided with bus tickets and all participants received a small honorarium.

Focus group discussions followed a sequence of guiding questions from a semistructured tool developed by the HSHC partnership as follows.

Semistructured Interview Guide

1. I would like to start by asking you what “healthy teeth” means for babies or very young children (under 5 years old). If I said that someone's child had healthy teeth, what would that mean to you?
  - Is it important for kids to have healthy teeth in your culture?
  - What do you think makes very young kids get cavities or decay in their baby teeth?
2. Do you think whether or not a child's baby teeth are healthy makes any difference to their overall health? If yes, ask how or in what ways.
3. Where did you learn how to take care of your babies' or young children's teeth?
  - Has anyone ever learned about dental care for babies or very young kids at any of the programs they attend?
  - Has anyone read any pamphlets or brochures about dental health for babies or young kids?
  - What do you think is the best way to get information out to parents and families about dental health for babies or young kids?
4. How do you take care of your babies' or young children's teeth?
  - Are there any specific practices that your culture does to keep children's teeth healthy?
  - What helps you keep your babies' or young children's teeth healthy?
  - Are there any things that make it hard for you to take care of your babies' or young children's teeth?
5. Does anyone here have children who have had problems with their teeth?
  - What kinds of problems did they have?
  - What did you do about it?
  - Does anyone here know any kids who have had dental surgery? If yes, ask what that was like for the kids and the families.
6. Is there one thing that somebody (anybody—government, health workers, family members, the people in this room, or anyone else you can think of) could do to help parents and caregivers take care of young children's teeth? What would it be?
7. Is there anything else that you would like to tell me about what we talked about today?

Questions of particular interest included what good oral health means for their child, their experiences with dental problems like ECC, and how they learned to care for their children's teeth. Another area of interest was whether there were any practices unique to their cultures relating to caring for young children's teeth. Additional probing questions were used as needed to elicit specific details or clarification. Notes and recordings from each focus group were transcribed verbatim and analyzed independently using thematic analysis by two members of the team. When analyzing the data, transcripts from each of the participant groups were examined independently, drawing out participants' responses to the overarching research questions. Themes that emerged in each cultural group were reported separately so that findings would be more practical to inform existing and future oral health promotion and research activities.

## 3. Results

A total of 40 parents and caregivers participated in this pilot study, including nine in the Aboriginal focus group and 14 in the Hutterite focus group. Eight were residents of the community where the focus group was held while the additional six resided at a different colony. The refugee group included 11 parents and caregivers. Participants originated from countries in Africa, the Middle East, and Western Asia including Chad, Congo, Ethiopia, Iraq, Morocco, Nigeria, and Somalia. Six people participated in the immigrant focus group and were from Africa and Western Asia including Congo, Eritrea, Nigeria, and Sudan.

## 4. Aboriginal Group

### 4.1. Definitions and Perceptions of Oral Health

Aboriginal participants described healthy teeth as being clean, free from decay, and not falling out. The majority agreed that baby teeth are important. Participants referred to a link between oral health and temperament, stating that

if they have a toothache, they're going to be all upset and miserable, crying, in pain and if they have a cavity, then they're going to be crabby. If they have healthy teeth, they won't be grouchy.

However, another participant felt that baby teeth are of little value as they are “going to fall out anyway.”

Two main risk factors for caries were identified. One was a mother's diet during pregnancy and the other was the use of bottles and bottle-feeding. One participant expressed,

“Everything you eat when you're pregnant, everything that goes in your mouth, your baby gets it”

Some participants believed that giving children a bottle at bedtime or naptime causes caries. While several participants had heard this before, a few stated that they did not believe this to be true.

Participants generally learned how to care for their children's teeth from their mothers, grandparents, and friends. One participant described how her grandparents tought her to use a facecloth and infant toothbrush to clean her babies' gums and teeth. Another indicated that she learned about using infant toothbrushes and toothpaste and the importance of antenatal oral health by attending a community-based Healthy Baby program.

Participants identified several barriers to adopting good family oral hygiene habits. This included uncooperative children, the cost and inability to purchase oral hygiene supplies, and lack of time. One participant expressed,

It's hard with my kids to get them to brush their teeth. I have to hold them there and brush for them. They do not like to brush their teeth. It only takes a couple of seconds, but it's a big deal.

Another said, “Mine are too lazy.”

Some caregivers indicated that they had little difficulty in getting their child to cooperate in brushing, though one parent noted that despite this her child still developed caries in her front teeth.

### 4.2. Participants' Experience with ECC

Three participants in the Aboriginal group had at least one child or family member who had experienced S-ECC and underwent dental surgery under general anesthesia (GA). One stated that her child's teeth had rotted before she reached the age of two because she did not have enough enamel and had surgery to remove these teeth. The mother of a three-year-old described the surgery experience as “*awful*.” Another stated that her niece had all her teeth removed when she was four years old.

Her teeth rotted really quickly. By the time she was three years old, her top and her bottom was just black, like on posters you see of tooth decay. That's how her teeth were.

Many of the parents indicated that they had difficulty in getting their children to see the dentist. For some, it was because they had been scared or hurt during previous dental encounters or feared needles. Unfortunately, one mother admitted that her son “has five cavities right now because he won't go to the dentist.” This fear of the dentist led two parents to agree that it might be better if the dentist were to simply use a gas to “just knock [their children] out.”

### 4.3. Cultural Practices as Related to Oral Health

Aboriginal participants shared information about traditional medicines and practices such as the use of herbal and traditional medicines when babies have rotten teeth. One had taken her child to a traditional healer because of the way “*the gums looked*” and had informed the dentist of this. However, participants suggested that before incorporating any traditional knowledge or medicines into programming and prevention activities, it is important to first seek permission from an elder to share knowledge and teachings.

### 4.4. Recommendations for Promoting ECOH

Sharing information on a one-to-one basis and making use of existing parental networks were described by participants as effective ways to promote ECOH within the urban Aboriginal community. It was suggested that front-line workers, such as public health nurses and dentists, begin making home visits. Participants also suggested that elders “talk to children in school about taking care of teeth and the [traditional] medicines.”

## 5. Hutterite Group

### 5.1. Definitions and Perceptions of Oral Health

Hutterite participants identified four factors they believed influenced oral health: oral hygiene, intake of junk food, use of fluoride, and genetics. Some participants felt that brushing and rinsing may be more important than a child's intake of candy and treats.

You can have a kid who does not eat candy and does not brush or a kid who eats lots of candy and brushes and the kid who eats a lot of candy will be better off.

Participants were not aware of colonies that fluoridate their drinking water, but noted that fluoride does occur naturally in the water of some colonies. Other colonies use water that has been treated by reverse osmosis to remove minerals and one participant wondered whether or not this might affect oral health as it removes fluoride from the water.

Genetics were also identified as possibly contributing to caries in Hutterite children. One mother pointed out that even in colonies where parents “*are making quite a bit of an effort*” to care for children's teeth, “*a lot of kids have to fill their teeth*.” Another stated that, while they did not remember ever brushing their teeth as children, they never had cavities and wondered if this might be due to genetics.

Parents and caregivers identified several obstacles to caring for their children's teeth. It was noted that, on several occasions, the children's temperaments hindered oral hygiene. Specifically, children were often too tired, grumpy, or simply unwilling to brush their teeth. Parents and caregivers also expressed difficulty in making the time to help or encourage their children to brush their teeth due to their own fatigue. Several parents in the group spoke about others giving candy to their children. One parent stated, “*I never give her candy, but she gets it from everybody else!*”

One caregiver acknowledged that she only cleans her children's teeth once a day, even though she knows it is recommended to wipe the teeth after each feeding. Her attempt to reduce the risk of decay was to give her baby water to drink, a practice that other parents in the group seemed to share. As one parent pointed out, it is important to clean babies' mouths because there are “something like 8 or 9 [sugar] cubes per cup” of breast milk, only “slightly less than juice.”

Participants admitted that it can be painful and traumatic for children when they have cavities, which can affect their quality of life: “if [children] have bad teeth, how can they eat?”

### 5.2. Participants' Experience with ECC

Two Hutterite participants had children who had dental surgery for S-ECC. One participant's four-year-old daughter had been complaining of a toothache so she took her to a dentist who “*found a whole mouth full of problems*.” She needed five teeth filled and another two removed.

I never want to go through it again… seeing her in all this pain and you cannot do anything at all. You just have to wait for this appointment. And it drives you crazy. And you're guilty. I took the blame. It's my fault. I did not take enough care of her teeth. Seeing her going into the operating room, they're going to put her to sleep and what if she never wakes up? And all of those things…

### 5.3. Cultural Practices as Related to Oral Health

Those who had learned about oral health through presentations in their communities were willing to share information with other family members. For instance, one passed along information to family members that you should not give a bottle to a child over one year of age. While participants felt empowered to share with family and friends, they indicated that they might feel uncomfortable about sharing information with others whom they did not know well.

 Participant 1: *I wouldn't dream of, if I see someone giving a baby a bottle, a two-year old, saying, “Do you know that's not healthy?”*
 Participant 2: *Of course not.*
 Participant 1: *If I know them—but not if I did not know them.*
 Participant 2: *They basically wouldn't have to listen to you.*
 Participant 1: *Well, it's none of our business. It's a personal preference.*
 Participant 3: *I might if I knew them a little—say do you know that this could cause this or that.*
 Participant 2: *But it's always better if they get it from somebody higher up.*
 Participant 1: *Like at a meeting or a workshop.*
 Participant 4: *That's non-confrontational.*

### 5.4. Recommendations for Promoting ECOH

Hutterite participants indicated that workshops are an effective way to share oral health messages. They appeared to value a “*personal, one-to-one connection*” style of learning. Oral health pamphlets and posters are displayed in the colony. Materials with both text and pictures were recommended as one parent stated, “nothing propels you more to try to help your child than to see the results of non-caring”, like “pictures of decayed teeth.”

However, they did say that “*if language is too high tech, nobody's going to read it*.” The community kitchen seems to be an established area for information sharing in Hutterite colonies.

Some caregivers mentioned that they sometimes obtain articles from the internet. They recommended strategies like a parenting blog, forum, or an email list serve or contact list as ways to disseminate information. Caregivers from this colony also indicated that public health nurses could take a more active role in providing information.

## 6. Newcomers: Immigrant and Refugee Groups

### 6.1. Definitions and Perceptions of Oral Health

Those in the immigrant group felt that good oral health meant the absence of swelling, pain, and broken teeth. One parent commented that “*if the first set of teeth starts bad then that will transfer to new [adult] teeth*.” Some in the refugee group felt that the health of baby teeth is important and explained that there is a relationship between overall health and healthy teeth. Another felt that baby teeth do not affect adult teeth.

Two refugee participants believed that genetic factors play a role in the process of decay, with one referring to the high occurrence of “*bad teeth*” in her family. The consumption of sweets, lack of oral hygiene, and the use of bottles were also identified as contributing factors in caries development.

Participants also mentioned the inability to control their children's intake of sweets at school, which makes it difficult for them to care for their children's teeth. Milk and dairy products were identified as good choices for children due to their calcium content. One mother from the immigrant group shared how she managed to curb her daughter's intake of sweets:

Sometimes you need to scare them. My daughter likes chocolate and sugar. When she has cereal, I give her a little sugar but she wants more. I tell her that if I give her more sugar, when I take her to the dentist, he'll remove all her teeth… Now, sometimes she says “Do not put sugar!”

Immigrant participants believed that regular visits to the dentist or doctor were important practices. However, those in the refugee group did not necessarily share this view, as one participant stated that children do not need to go to the dentist unless they are experiencing dental problems.

One immigrant mother mentioned how the dentist recommended that she give her daughter a cup rather than a bottle as her daughter's teeth had “*turned black*.” She said the “*bottle is not good for teeth*.” Other participants agreed that children should start using a cup at an early age instead of bottles.

Parents and caregivers also shared information regarding oral hygiene practices at home. They described cleaning their babies' gums and tongue using a cloth, warm water and salt, baking soda, glycerin, or cotton wool. The majority of participants indicated they had first learned about oral health care from family members and friends and later from medical practitioners in their home countries. As one stated,

“I do with my daughter the same my mom did with me.”

### 6.2. Participants' Experience with ECC

Children of participants from the immigrant focus group were reported to have had few dental problems. However, one child did develop caries involving the primary maxillary incisors. The mother described her experience:

[The] family doctor, when my child's tooth was a little black, he told me to go to dentist and gave address—but no other information. The dentist said there's nothing too bad about the teeth—it's just the colour. And when her new teeth come out, they'll be better. He said to brush all the time and I do not have to feed her by the bottle. When she was small, I gave her most of the time a bottle. That's why she had the problem. So I have to feed her by the cup and you have to clean always her teeth.

### 6.3. Cultural Practices as Related to Oral Health

Participants in both groups spoke about the practice of using a twig from a specific tree to clean teeth, stating that it has the additional benefit of being natural and chemical-free. They referred to this twig as a “*sewak”* and reported that the plant has “*lots of benefits for your teeth*.” The twig is reportedly very effective: “*Sometimes a brush won't get everything, but that one will take everything off.”* Some bring these twigs back when they return from visits to their homeland. Another added that the twig can also be purchased locally.

Participants in the refugee group discussed the importance of hygiene to the Muslim faith. As one participant stated,

It's part of the obligation. As part of Islam, we pray 5 times in the day. It is most recommended that you brush your teeth. There is a saying from our prophet that if I would have told any human being that these are the obligations that you must do, I would have encouraged them to clean their teeth five times a day. He did not say it's a must for you—it's a very strong recommendation that it is very important.

### 6.4. Recommendations for Promoting ECOH

Participants suggested that oral health promotion activities could be delivered through existing programs, classes, daycares, schools, and organizations in which parents are already involved (such as EAL classes or programs for moms and tots).

That's a good reason to use community centres—they can bring parents out, tell them what you want to say, what they need to do. For people who do not understand the language, it's better for them to see it with their eyes.

Some indicated that they would appreciate getting information from a healthcare provider with experience and knowledge whom they could easily trust. One suggested that family doctors could distribute oral health information during immunization appointments. Others felt that basic information could be delivered by laypeople. People from their own cultural community could be trained to pass on this information. There was general agreement that some refugee caregivers might prefer “*someone who is like them*” or who knows their language.

## 7. Discussion

The purpose of this pilot study was to gain an initial understanding of views on ECOH that may assist in shaping effective and appropriate culturally proficient promotional activities and materials targeting specific “communities” within an increasing diverse population.

Even though the intent of these pilot focus groups was not to contrast findings between the different cultural groupings, it was interesting that there were some differences and apparent similarities. For instance, when asked what contributes to caries in young children, participants in the Aboriginal group identified bottles and bottle-feeding along with prenatal diet as being important while Hutterite participants identified a lack of fluoride in the drinking water, junk food, and genetics. Meanwhile, newcomer participants mentioned sweets, a lack of oral hygiene, and genetics. One apparent similarity between some of the groups related to barriers to regularly cleaning their children's teeth was seen as both participants in the Aboriginal and Hutterite groups mentioned a lack of time as well as their children's temperament and uncooperativeness. With regard to promoting ECOH each group mentioned the importance of reaching parents and making personal connections but offered unique suggestions ranging from including Aboriginal elders to share traditional knowledge, the use of workshops and health-education materials with the Hutterites, and using laypeople in newcomer communities to including oral health messages in existing programs providing assistance to these families.

Each focus group yielded useful suggestions on how to possibly promote oral health and engage members of their cultural community. For instance, Aboriginal participants discussed at length the role of elders. Two specific issues were identified, namely, seeking permission from an elder to incorporate traditional medicine or knowledge into programming and the elder's actual role in information sharing. These findings are consistent with those of a study examining cultural factors affecting children's oral health, which found that elders and their wisdom were highly respected [17].

The Hutterite focus group elicited information not discussed in the other groups. They discussed concerns of passing on information to strangers and the importance of using a nonjudgmental approach as some felt guilty that their child required dental surgery. They felt that appropriate methods included the use of pamphlets, posters, and e-mail updates. This resembles a health-education model rather than health promotion and community development approaches and is not recommended for groups with low literacy levels, language barriers, or limited access to computers. Hutterite communities have a unique lifestyle as they live communally with community ownership of most goods. Communal living allows for less control over some aspects of living as compared to other groups, as is evident with shared meals, dress, and lack of individual finances [30]. This lifestyle may impact their access to oral hygiene supplies and dental care. As decision-making occurs at the community elder level, efforts need to be directed to educating and building relationships with community leaders. It is important to note that women in Hutterite culture play a key role in making decisions about health [11, 30]. We previously reported that Hutterite mothers had a highly accurate view of their children's oral health [11].

Both Hutterite and refugee participants believed that genetics play a role in ECC development. While there is a proven increase in dental agenesis in the Hutterite population, presently there is no literature to substantiate a genetic predisposition to caries with this group [31]. This belief is likely based on the fact that some genetically associated diseases, such as muscular dystrophy and cystic fibrosis, are more prevalent in or exclusive to the Hutterite population [32, 33]. Some genetic conditions do affect enamel and dentin formation, which can decrease host resistance to caries (e.g. amelogenesis imperfecta). However, there is now emerging evidence supporting a genetic predisposition to caries in some populations [34–36]. The belief that hereditary factors contribute to caries is not exclusive to our study, as these views were also held by Latino immigrant caregivers in another investigation [24]. Regardless of the role that genetics play, it is important to increase parental awareness of the numerous factors involved in caries development so that they can minimize their children's caries risk.

Interestingly, some participants in the Aboriginal group did not believe that putting children to sleep with a bottle could cause caries. This was surprising, as bottle misuse is a highly cariogenic practice. Other reports have suggested that some parents may not understand this and may routinely give their infants and toddlers bottles at bedtime [23, 37]. In a recent qualitative study, nurses reported that parents often do not associate bottle-feeding with caries [38]. Our findings also suggest that there may be some misconceptions about general infant feeding that require clarification. For instance, participants from the Hutterite group stated that breast milk is high in sugar. While breast milk does contain a certain amount of natural sugar, breast milk itself is not cariogenic [39]. A recent review suggests that there is inconclusive evidence to support a relationship between breastfeeding and ECC [40]. However, while some studies have reported that breastfeeding may be protective against caries [41] other studies have reported that prolonged breastfeeding and nocturnal breastfeeding may increase the risk [42]. The Canadian Dental Association's recent position statement on breastfeeding supports this practice but emphasizes the importance of regular oral hygiene once primary teeth begin to erupt [43].

Aboriginal participants received most of their oral health information from mothers and grandmothers. Therefore, it may be important to involve parents and grandparents in oral health promotion activities to equip them with essential oral-health-related information that they can then pass on to younger generations. A move towards family-centred care (which encourages the involvement of all members of a patient's circle, both familial and social) would assist in meeting the needs of this group [44]. In our study, only one participant received oral health messages from a health professional. This was surprising, as the group identified public health nurses as a possible messenger of oral health information.

Immigrant participants possessed a good level of understanding about ECOH and few had children who developed ECC. This may be in part due to what is called the “healthy immigrant effect,” which suggests that the healthiest are more likely to migrate and be granted residence in another country [45]. Participants in this focus group held differing opinions about who should deliver information to members of their community. While some felt that professionals would be best as they “trust” them, others believed that lay workers in the community would be better suited to promote ECOH. This has been shown to be effective in the Vietnamese community in British Columbia [14].

Several common themes emerged from the different pilot focus groups. For instance, participants from each group identified that the difficulty in cleaning their children's teeth and limiting sugar intake were challenges to keeping their children's mouths healthy. Similar findings were also reported in a recent study involving African newcomers to Canada as they expressed concern over their inability to keep their children from eating sugar and candies and fighting with their children to brush their teeth [21].

Refugee participants believed that few of their children had dental issues and suggested that children really only need to visit the dentist when they experience a dental problem or toothache. Similarly, another report has suggested that the perceived need for dental care may be low among African newcomers as they mainly rely on their own assessments, toothaches, and advanced signs of caries to indicate the need for dental care rather than the established early warning signs of ECC [21]. Additional evidence supports these findings, as certain groups have been found to seek dental care only after their children begin to experience pain [46]. Seeking preventive dental care may not be the cultural norm [21, 46].

The refugee group discussed the influence of religion on oral health and hygiene. Many participants identified as Muslim said that performing oral hygiene is part of Islam. However, focus groups with a similar population of Canadian newcomers have suggested that oral hygiene may not be a priority, as they believe that oral health is ultimately dictated by God's will [21]. Perhaps the involvement of religious institutions and leaders may be a worthwhile avenue to explore for continuing work with this population.

Language is key to effective and safe communication and therefore must play a critical role if ECOH is to be effectively tailored to specific populations. Participants suggested that using individuals from their own cultural group to deliver oral health messages would be effective. Language barriers have a larger influence on how one successfully interacts with the health care system than cultural beliefs [47]. Language is affected by cultural and historical context and is “often about sharing and validating realities” [48]. Given the obvious language barriers that exist for newcomer populations, participants in the immigrant and refugee groups also suggested that providing visual information and resources may be useful in sharing key messages about ECOH. As rates of immigration continue to grow, cultural groups are less likely to have access to health professionals who share the same beliefs and understandings of health and disease, language, and experiences [49]. “Linguistically appropriate care” can be achieved when a provider shares an understanding of the experiences of the community [48]. Perhaps the use of interpreters at dental care appointments can help to pass along oral health messages. This service exists in some community-based dental programs in the Winnipeg region.

For practitioners to provide “culturally responsive care” they require awareness of cultural beliefs and practices while recognizing that care still needs to be provided based on an assessment of the individual [49]. Health promotion workers should continue to learn about distinct cultural groups while recognizing that communication and individual beliefs will still have an impact on knowledge acquisition and behavioural change. This approach will help shape health promotion activities and develop prevention strategies targeted to unique at-risk groups. If we are going to reduce the impact of ECC on these groups, we must ensure that preventive strategies are adapted as necessary and incorporate their suggestions.

There are several limitations to our pilot work. Due to our sampling approach, the findings are not generalizable to the entire communities participating in our study as these findings may not be reliable and reproducible if more representative samples were recruited. Further, those agreeing to participate may have been those with a greater appreciation and awareness of oral health. Participation was not restricted to only parents and caregivers of children who were affected by ECC, which may have resulted in an overrepresentation of those whose children were actually in good dental health. The small numbers of participants and the pilot nature of this work also prohibit comparisons between groups. Language issues proved to be a large hurdle in the focus group process, as several participants spoke English as an additional language. This was particularly evident in both focus groups with parents and caregivers who were refugees or other immigrants. While all individuals in the immigrant focus group spoke English well, the majority of participants in the refugee group had limited English skills and relied on other participants to translate for them. The reliance on these individuals as translators constituted another source of error, as the information obtained by researchers was, in a sense, passed through an intermediate party which had “interpretive control.” The interpreters had control over what they communicated as the content and meaning of their language peers' responses. While the immigrant and refugee focus groups were somewhat heterogeneous in terms of country of origin, it can be argued that all participants in each respective group shared similarity as they self-identified as being either an immigrant or refugee. Participants in this study may have already had some understanding of ECC through exposure to the HSHC initiative or other resources. Regardless, the information obtained during these focus group sessions is extremely valuable and provides useful insight into the best ways to promote ECOH amongst these at-risk populations.

The HSHC partnership understands that meaningful community development requires that attention be paid to cultural proficiency for meaningful community engagement, the development of interventions, oral health promotion, and health education. That is why this pilot work was undertaken. Culturally and linguistically proficient approaches must be developed for at-risk communities if they are to fully participate in prevention and promotional activities [50]. Developing culturally proficient and therefore relevant approaches to oral health promotion and caries prevention requires an understanding of diversity. Cultural proficiency can be enhanced by increasing awareness of the views and beliefs of cultural groups.

This pilot work will certainly help to inform our further qualitative and quantitative research and outreach activities with these different groups, especially immigrants and refugees to Manitoba, Canada. There is a growing need for further qualitative investigation with larger samples of parents, especially those whose children have experienced ECC, to gain their perspectives. Larger sample sizes would also assist in drawing comparisons between different cultural groups. This would also assist in the development of questions for use in survey instruments and caries-risk assessment tools for these cultural groups. Since little is known about the true oral health status of refugee and immigrant newcomers to Manitoba, baseline studies on the prevalence of ECC and associated risk factors are warranted. At the present time we are using these findings to assist us in developing pictorial-based ECOH promotion materials for newcomer populations.

## 8. Conclusion

These pilot focus group sessions were useful in identifying potential barriers to ECOH, sources of oral-health information, oral health-related misconceptions, and how to best reach each community with ECOH messages. Caregivers identified several barriers to maintaining ideal early childhood oral health including the child's temperament, finances, and inability to control sugar intake. Each group appeared to have a reasonable understanding of early childhood oral health. However, both religion and genetics were found to influence the perception of oral health in some groups. Misconceptions regarding breast milk and bottle use were present. while participants from the refugee group believed that dental visits were only necessary if dental pain or problems were experienced.

Each group proposed strategies to improve oral health promotion. One-on-one discussions, use of parental networks, and the use of laypeople from similar cultural backgrounds were suggested as ways to promote oral health. The immigrant and refugee group placed emphasis on the use of visuals for those with language barriers while the Hutterite participants recommended a more traditional health-education focused approach.

The findings from this paper have provided some initial insight into the oral-health-related knowledge and beliefs of these high-risk cultural groups. These insights will help to inform planning of ECC prevention and research strategies, which can be tailored to specific populations.

## References

[1] Schroth RJ, Harrison RL, Moffatt MEK (2009). Oral health of indigenous children and the influence of early childhood caries on childhood health and well-being. *Pediatric Clinics of North America*.

[2] Peretz B, Ram D, Azo E, Efrat Y (2003). Preschool caries as an indicator of future caries: a longitudinal study. *Pediatric Dentistry*.

[3] Al-Shalan TA (1997). Primary incisor decay before age 4 as a risk factor for future dental caries. *Pediatric Dentistry*.

[4] Drury TF, Horowitz AM, Ismail AI, Maertens MP, Rozier RG, Selwitz RH (1999). Diagnosing and reporting early childhood caries for research purposes. *Journal of Public Health Dentistry*.

[5] American Academy of Pediatric Dentistry (2010). Definition of Early Childhood Caries (ECC). *Pediatric Dentistry*.

[6] Peressini S, Leake JL, Mayhall JT, Maar M, Trudeau R (2004). Prevalence of early childhood caries among First Nations children, District of Manitoulin, Ontario. *International Journal of Paediatric Dentistry*.

[7] Schroth RJ, Cheba V (2007). Determining the prevalence and risk factors for early childhood caries in a community dental health clinic. *Pediatric Dentistry*.

[8] Schroth RJ, Moore P, Brothwell DJ (2005). Prevalence of early childhood caries in 4 Manitoba communities. *Journal of the Canadian Dental Association*.

[9] Schroth RJ, Smith PJ, Whalen JC, Lekic C, Moffatt MEK (2005). Prevalence of caries among preschool-aged children in a Northern Manitoba Community. *Journal of the Canadian Dental Association*.

[10] The First Nations Information Governance Centre (2012). *The First Nations Information Governance Centre, First Nations Regional Health Survey (RHS) Phase2 (2008/10) National Report on Adults, Youth and Children Living in First Nations Communities*.

[11] Schroth R, Dahl P, Haque M, Kliewer E (2010). Early childhood caries among Hutterite preschool children in Manitoba, Canada. *Rural and Remote Health*.

[12] Werneck RI, Lawrence HP, Kulkarni GV, Locker D (2008). Early childhood caries and access to dental care among children of Portuguese-speaking immigrants in the city of Toronto. *Journal of the Canadian Dental Association*.

[13] Weinstein P, Harrison R, Benton T (2006). Motivating mothers to prevent caries: confirming the beneficial effect of counseling. *Journal of the American Dental Association*.

[14] Harrison R, Wong T, Ewan C, Contreras B, Phung Y (1997). Feeding practices and dental caries in an urban Canadian population of Vietnamese preschool children. *Journal of Dentistry for Children*.

[15] American Academy of Pediatric Dentistry (2010). *Guideline on Caries-Risk Assessment and Management for Infants, Children, and Adolescents*.

[16] Kay E, Locker D (1998). A systematic review of the effectiveness of health promotion aimed at improving oral health. *Community Dental Health*.

[17] Hilton IV, Stephen S, Barker JC, Weintraub JA (2007). Cultural factors and children’s oral health care: a qualitative study of carers of young children. *Community Dentistry and Oral Epidemiology*.

[18] Selikowitz HS (1994). Acknowledging cultural differences in the care of refugees and immigrants. *International Dental Journal*.

[19] Scrimshaw SC (2003). Our multicultural society: implications for pediatric dental practice. *Pediatric Dentistry*.

[20] Kitzinger J (1995). Introducing focus groups. *British Medical Journal*.

[21] Amin M, Perez A (2012). Is the wait-for-patient-to-come approach suitable for African newcomers to Alberta, Canada?. *Community Dentistry and Oral Epidemiology*.

[22] Wong D, Perez-Spiess S, Julliard K (2005). Attitudes of chinese parents toward the oral health of their children with caries: a qualitative study. *Pediatric Dentistry*.

[23] Schroth RJ, Brothwell DJ, Moffatt MEK (2007). Caregiver knowledge and attitudes of preschool oral health and Early Childhood Caries (ECC). *International Journal of Circumpolar Health*.

[24] Horton S, Barker JC (2008). Rural Latino immigrant caregivers’ conceptions of their children’s oral disease. *Journal of Public Health Dentistry*.

[25] Health Canada (2001). *‘Certain Circumstances’. Issues in Equity and Responsiveness to Health Care in Canada*.

[26] Macintosh AC, Schroth RJ, Edwards J, Harms L, Mellon B, Moffatt M (2010). The impact of community workshops on improving early childhood oral health knowledge. *Pediatric Dentistry*.

[27] Schroth RJ, Morey B (2007). Providing timely dental treatment for young children under general anesthesia is a government priority. *Journal of the Canadian Dental Association*.

[28] Schroth R, Edwards J, Moffatt M (2010). *Healthy Smile Happy Child: Evaluation of a Capacity-Building Early Childhood Oral Health Promotion Initiative*.

[29] Laverack G, Labonte R (2000). A planning framework for community empowerment goals within health promotion. *Health Policy and Planning*.

[30] Hofer S (1998). *The Hutterites. Lives and Images of a Communal People*.

[31] Mahaney MC, Fujiwara TM, Morgan K (1990). Dental agenesis in the Dariusleut Hutterite Brethren: comparisons to selected caucasoid population surveys. *American Journal of Physical Anthropology*.

[32] Klinger K, Horn GT, Stanislovitis P, Schwartz RH, Fujiwara TM, Morgan K (1990). Cystic fibrosis mutations in the Hutterite Brethren. *American Journal of Human Genetics*.

[33] Armistead J, Khatkar S, Meyer B (2009). Mutation of a gene essential for ribosome biogenesis, EMG1, causes Bowen-Conradi syndrome. *American Journal of Human Genetics*.

[34] Kulkarni GV, Chng T, Eny KM (2013). Association of GLUT2 and TAS1R2 genotypes with risk for dental caries. *Caries Research*.

[35] Zeng Z, Shaffer JR, Wang X (2013). Genome-wide association studies of pit-and-fissure- and smooth-surface caries in permanent dentition. *Journal of Dental Research*.

[36] Hughes T, Bockmann M, Mihailidis S (2013). Genetic, epigenetic, and environmental influences on dentofacial structures and oral health: ongoing studies of Australian twins and their families. *Twin Research and Human Genetics*.

[37] Weinstein P, Troyer R, Jacobi D, Moccasin M (1999). Dental experiences and parenting practices of Native American mothers and caretakers: what we can learn for the prevention of Baby Bottle Tooth Decay. *Journal of Dentistry for Children*.

[38] Arora A, Bedros D, Bhole S (2012). Child and family health nurses’ experiences of oral health of preschool children: a qualitative approach. *Journal of Public Health Dentistry*.

[39] Erickson PR, Mazhari E (1999). Investigation of the role of human breast milk in caries development. *Pediatric Dentistry*.

[40] Salone LR, Vann WF, Dee DL (2013). Breastfeeding: an overview of oral and general health benefits. *Journal of the American Dental Association*.

[41] Schroth RJ, Halchuk S, Star L (2013). Prevalence and risk factors of caregiver reported Severe Early Childhood Caries in Manitoba First Nations children: results from the RHS Phase 2 (2008–2010). *International Journal of Circumpolar Health*.

[42] Valaitis R, Hesch R, Passarelli C, Sheehan D, Sinton J (2000). A systematic review of the relationship between breastfeeding and early childhood caries. *Canadian Journal of Public Health*.

[43] (2013). Does breastfeeding increase risk of early childhood caries?. *Journal of the Canadian Dental Association*.

[44] Moffatt MEK, Cook C (2005). How can the health community foster and promote the health of Aboriginal children and youth?. *Paediatrics and Child Health*.

[45] WRHA Research and Evaluation Unit (2010). *Part One: Setting the Context. Health of Immigrants and Refugees in the Winnipeg Health Region: A Community Health Assessment Resource for Health Services Planning*.

[46] Naidu R, Nunn J, Forde M (2012). Oral healthcare of preschool children in Trinidad: a qualitative study of parents and caregivers. *BMC Oral Health*.

[47] Bowen S (2001). *Language Barriers in Access to Health Care*.

[48] Putsch R (2003). *Reflections on the CLAS Standards: Best Practices, Innovations and Horizons*.

[49] Bowen S (2008). Providing care in a changing society: what do health care providers really need to know about cultural diversity?. *Canadian Journal of Dental Hygiene*.

[50] Winnipeg Regional Health Authority (2011). *Framework for Action. Cultural Proficiency and Diversity*.

